# Beyond Pattern Recognition: Call for Functionally Aware AI for Anatomical Illustration

**DOI:** 10.2196/92700

**Published:** 2026-08-20

**Authors:** Agata Maria Kawalec-Rutkowska, Marian Simka

**Affiliations:** 1 Department of Anatomy, Institute of Medical Sciences, University of Opole, Oleska 48, Opole, Opole Voivodeship, 45040, Poland, 48 607572642

**Keywords:** artificial intelligence, AI, anatomical illustration, medical education, spatial reasoning deficit, intellectual property

## Abstract

This paper critically assesses the role of generative AI in anatomical illustration, identifying fundamental barriers that currently preclude AI from replacing human medical illustrators. Despite the promise of unprecedented efficiency, contemporary models exhibit persistent anatomical inaccuracies and “hallucinations” of nonexistent structures—flaws stemming from statistical pattern-matching rather than genuine anatomical understanding. These systems further lack pedagogical intent, clinical context, and the capacity for deliberate visual judgment, while raising unresolved ethical and copyright concerns regarding training data. Although a specialized AI for this purpose is theoretically feasible, its development as a standalone goal remains economically nonviable given the niche nature of the profession. Rather than replacing human illustrators, AI’s future role will be augmentative, with the requisite anatomical intelligence likely emerging as a byproduct of broader advances in clinical applications such as surgical planning and personalized medicine. For AI-generated imagery to become educationally and clinically reliable, it will require rigorous human supervision, curated gold standard datasets, and a foundation of genuine anatomical comprehension.

## Introduction

Since the pioneering work of Renaissance masters like Leonardo da Vinci and Andreas Vesalius, anatomical illustration has been a cornerstone of medical science and education. Da Vinci’s meticulous, fluid sketches and Vesalius’ groundbreaking, systematic representations in *De Humani Corporis Fabrica* did more than just document the human form; they transformed our understanding of it, establishing a visual language that bridged empirical observation with pedagogical clarity. For centuries, this tradition of hand-rendered accuracy has been indispensable, allowing generations of students to visualize the intricate architecture of the body [1,2].

Today, AI emerges as a potential new frontier, promising to generate intricate anatomical models with unprecedented speed and scale. The seamless integration of AI-powered anatomical illustrations would significantly facilitate medical education at both the undergraduate and postgraduate levels. In particular, the ability to present students with high-quality, error-free drawings that accurately depict anatomical variants would greatly enhance instruction across many specialties [3,4]. Contemporary textbooks and atlases typically illustrate only “standard” anatomy; to showcase clinically relevant variants, educators must search for them in scattered articles—a time-consuming process often complicated by authorship restrictions. Moreover, these resources often underrepresent diverse populations, with illustrations disproportionately reflecting a narrow demographic—most commonly young adult males of a single ethnic background—thereby limiting learners’ preparedness for treating patients of different sexes, ethnicities, and age groups [5,6]. Similarly, existing anatomical illustrations usually present a structure from only one or at most a few perspectives, making it difficult to augment clinical reasoning with visuals that fully capture spatial relationships. Such illustrations—those that emphasize anatomical variability and spatial relationships—are not easily accessible today. The high cost and time required to create such educational tools on demand, if a human illustrator were the author, put them out of reach for the majority of medical universities. AI-powered illustration tools could overcome these limitations by generating variant-rich, multiperspective, and demographically inclusive imagery on demand, thereby bridging the gap between anatomical knowledge and clinical application. However, this promise is tempered by significant barriers.

This paper presents an expert viewpoint, based primarily on the authors’ practical experiences with AI-generated anatomical artworks in undergraduate anatomical courses, as well as on current published evidence regarding this topic. To inform this perspective, we conducted a literature search and performed a critical analysis of the possible sources of AI incapabilities in this domain. In addition, we went beyond formal analysis by drawing on our personal experiences in teaching anatomy—specifically, guiding students to transform textbook and atlas knowledge into clinical reasoning [7]. This paper will therefore examine the critical problems encountered by current AI applications in generating anatomical illustrations and assess the feasibility of overcoming these challenges in the future.

## Current Limitations of AI in Anatomical Illustration

### Problems Related to Current Generative AI Capabilities

Despite the promising potential of AI, its application in anatomical illustration is fraught with significant challenges that currently limit its reliability for precise medical and educational use. Current generative AI systems are fundamentally unsuited to replace human medical illustrators in creating educational anatomical images due to a core disconnect: they operate on statistical patterns, not conceptual understanding. Whether future systems might overcome this limitation remains an open question. Models like DALL-E and Midjourney are sophisticated pattern-matching systems that associate text prompts with visual data, but they possess no knowledge of functional anatomy, 3D spatial relationships, or physiological logic. In essence, current AI models generate a visually compelling collage of patterns, rather than a coherent, functional anatomical system [8-13]. As demonstrated by Moin et al [8] in their assessment of corneal transplant illustrations and by Davis et al [9] in a neurosurgical context, AI models frequently generate images with critical errors. These can range from the misplacement of structures and incorrect spatial relationships to the fabrication of entirely nonexistent anatomical features, a phenomenon known as “hallucination.” For instance, Kumar et al [10] highlighted how AI struggled to accurately depict the nuanced pathophysiology of conditions like Horner syndrome, producing visually compelling but scientifically incorrect images. This inherent unpredictability poses a direct threat to the foundational principle of accuracy in medical science [11]. Importantly, a hallucinated blood vessel or an incorrectly positioned nerve is not a stylistic choice. It is a critical failure that can mislead a student or misinform a surgeon [11,14-19]. This AI’s inability to create accurate and educational images stems from a problem more fundamental than a simple lack of training data. Critically, medical illustration is not merely the photographic reproduction of an anatomical specimen. This distinction is evident when comparing illustrated anatomical atlases to photographic ones; the former are often superior for education, even at the cost of photorealistic detail. In a masterful medical illustration, such as those by Frank H Netter, the artist makes deliberate pedagogical choices: key structures are emphasized and clarified, while less relevant details are subdued or sketched. This hierarchy of information, designed to guide the viewer’s eye and understanding, is the core of its educational value [20]. This essential, goal-oriented design currently cannot be achieved without the illustrator’s deep, firsthand experience derived from real contact with the human body—both in the dissection lab and the operating room—forging a cognitive link between form, function, and clinical relevance. Current AI models, trained on pixels and patterns, still lack the clinical judgment and intentionality to make these decisions reliably. They cannot yet discern what is important to teach without expert guidance.

While current generative AI is unreliable for anatomical illustration, the creation of a specialized, accurate tool is not beyond its future capabilities. The significant barriers hindering AI’s adoption in anatomical illustration stand in sharp contrast to its rapid and transformative integration into other creative and technical fields. In domains like marketing, conceptual art, and graphic design, AI image generators have become powerful, widely accepted tools. In these areas, an AI-generated image is valued for its aesthetic impact and its ability to rapidly visualize a mood or a concept. This conceptual flexibility allows human artists to use AI as a “copilot” for brainstorming and rapid prototyping, dramatically speeding up workflows [21-25].

This stands in direct opposition to the nonnegotiable demands of medical education. As emphasized by Zoltie [26] and Adams and Erolin [27], a human medical illustrator does not merely replicate anatomy but curates, emphasizes, and simplifies complex information to guide a learner’s focus and enhance comprehension. While generative AI tools lack intrinsic intentionality, they are nonetheless capable of producing goal-directed outputs. A similar concern arose in natural language generation, where such goal-directed capabilities were initially thought to be beyond AI—an assumption later proven incorrect. In the case of anatomical art, similarly pedagogically structured outputs are achievable. Thus, the current limitations of AI-generated anatomical illustrations appear to stem less from an inherent inability to exercise judgment and more from the absence of sufficiently grounded anatomical, spatial, and clinical data with which to train these models. This deficiency is exacerbated by the nature of traditional medical knowledge itself. Current anatomical textbooks and atlases, at their core, provide 1D (textual) or 2D (illustrational, cross-sectional) information [7]. Feeding this existing corpus to a standard AI in its current form would be inefficient, as it would risk perpetuating the same 2D pattern-matching limitations, making it difficult to infer the true spatial relationships and functional dynamics of living anatomy. Of note, even the most accurate and high-quality AI-generated illustrations cannot fully replace education based on cadaver dissections and the study of cadaveric prosection specimens, which remain essential for developing 3D spatial understanding and tactile appreciation of the human body [28,29].

### Problems Related to Ethical and Intellectual Property Issues

Beyond factual errors, significant ethical and legal dilemmas remain unresolved. The training of generative AI models relies on vast datasets of existing images, often scraped from the internet without the explicit consent of the original creators. As noted by Cornwall et al [30] and Jaillant and Aske [31], this raises profound questions about copyright infringement and the intellectual property rights of medical illustrators whose work is used without attribution or compensation. Furthermore, the “black box” nature of many AI systems, as discussed by Chen et al [14], creates a problem of explainability. If an AI generates an erroneous illustration, it is often impossible to trace the logical pathway that led to the mistake, undermining scientific accountability [32].

Similarly, the materials used to train AI systems must meet rigorous ethical standards: body donors should provide explicit written consent for the use of their bodies in AI training, and the same principle should apply to medical images drawn from hospital datasets such as computed tomography (CT) and magnetic resonance imaging (MRI) scans [33]. Furthermore, existing published medical illustrations must be protected from unauthorized use for AI training; alternatively, their authors or copyright holders should receive appropriate financial compensation for such use [32].

## A Path Toward AI-Driven Anatomical Illustration

### Problems to Overcome

Significant technical and logistical hurdles should be overcome to move beyond general-purpose models. The most promising solution lies in developing highly specialized “expert AIs” trained not on the chaotic internet, but on meticulously curated, gold standard datasets. These datasets would comprise verified sources like textbook illustrations (eg, Netter’s illustrations), labeled cadaver photos, and annotated 3D models, effectively teaching the AI a prior example of correct anatomy and eliminating its current tendency to create inaccurate averages. A practical implementation of this is fine-tuning existing powerful models on this specialized data, steering their inherent capability for rendering and style toward anatomical precision. Even more powerful could be a hybrid approach, where AI would be integrated with a rule-based 3D anatomy atlas. In this model, the software would generate a perfectly accurate 3D structure, and the AI’s role would be limited to applying requested styles, angles, and lighting—bypassing its weakness in 3D reasoning and leveraging its strength in visualization. This development cycle could be refined through reinforcement learning from human feedback, where expert illustrators and surgeons would continuously rate outputs, training the model to prioritize accuracy. These challenges are not trivial. They involve the immense cost and effort of dataset curation, navigating copyright issues, and the computational expense of training. Yet, this is not merely theoretical. Research institutions and medical technology startups are already actively fine-tuning models on biomedical data—including 3D images, which are similarly structured to anatomical illustrations. Such attempts are proliferating across clinical medicine, with numerous examples where AI is being adapted to tackle complex diagnostic and planning tasks. Current research is actively bridging the gap between AI’s pattern-matching prowess and the nuanced demands of clinical medicine [34-38]. One pertinent example concerns management of acute stroke, specifically the assessment of the penumbra—the brain tissue at risk of irreversible ischemic damage. While algorithm-based software, such as the rapid processing of perfusion and diffusion (RAPID), remains the gold standard in stroke management [39], a number of companies are now developing AI-driven tools for this purpose. Although these attempts are still suboptimal compared to established solutions, significant progress is being made in this area. Of note, financial barriers are likely of minor significance here, as existing software for stroke assessment is already quite costly, and the end users—hospitals—are eager to pay for solutions that can improve patient outcomes and streamline critical care workflows [40-42].

### AI-Driven Anatomical Illustration as Part of a Bigger Task

While the prospect of an AI capable of generating accurate anatomical illustrations is not beyond the realm of possibility, the standalone economic case for developing such a tool is weak. The profession of medical illustration is highly specialized and niche, meaning that the market for a tool designed solely for this purpose is likely too small to justify the immense cost and effort required to create a specialized, gold standard dataset and model. The necessary capabilities for anatomical illustration are far more likely to be acquired by AI as a byproduct of solving larger, more financially viable medical challenges. We are already witnessing this foundational work in other domains. In radiology [43-46] and epidemiology [47-49], AI excels at finding hidden patterns in vast datasets—whether tracking disease spread or identifying a tumor in a CT scan—tasks where its pattern-recognition prowess offers a clear advantage. Yet, many other medical specializations remain beyond AI’s current reach precisely because they require a synthesis of multidimensional knowledge. Current AI systems cannot yet replicate this integration, in part because they lack a fundamental, 3D understanding of the human body.

A successful approach would require a paradigm shift, not merely more data. Even the ambitious inclusion of massive, real-world patient data from national-scale medical repositories, as recently attempted in China [50], may yield diminishing returns. Such approaches primarily offer more data, not deeper comprehension. The true breakthrough will require moving beyond mere data correlation to building a foundational, causal model of the human body. This necessitates an AI that does not just process information but actively “learns” human anatomy in 4D—understanding the dynamic, 3D relationships between structures and their integrated function in health and disease. Without this core anatomical intelligence, AI’s diagnostic and therapeutic insights will remain reactive, confined to patterns of the past. To excel in these areas, an AI would need to be built upon a foundational, interactive 3D model of human anatomy—a digital twin that understands biomechanics, physiological pathways, and spatial relationships. It would learn from dynamic sources, like surgical videos, real-time sensor data, and 3D medical scans, moving beyond static textbooks and atlases. Creating such a model is a monumental task, but one with a compelling economic and clinical rationale that a niche illustration tool would lack. Once this robust, functionally aware anatomical intelligence exists, the ability to generate correct and educational illustrations could emerge as a natural byproduct. Consequently, the future of AI in anatomical art is inextricably linked to its evolution in core clinical reasoning.

## Conclusions

Despite its promise, AI’s role as an anatomical illustrator is currently impeded by fundamental obstacles: persistent anatomical inaccuracies and hallucinations, unresolved ethical and copyright issues surrounding training data, and a lack of genuine 3D and pedagogical understanding—a deficiency rooted in the fact that current AI tools lack intrinsic intentionality, operating on statistical patterns rather than deliberate educational or spatial reasoning. Overcoming these barriers will require a deliberate shift from general-purpose models toward specialized, expert-driven systems trained on curated, gold standard datasets, potentially integrated with rule-based 3D anatomy platforms. In the future, AI is unlikely to replace human medical illustrators but will instead serve as a sophisticated tool that augments their expertise. Such capabilities will likely emerge as a byproduct of AI’s broader evolution in clinical domains like surgical planning and personalized medicine, where robust anatomical understanding is a prerequisite.

## Abbreviations

CT: computed tomography
MRI: magnetic resonance imaging
RAPID: rapid processing of perfusion and diffusion
